# On the Origin of Candida auris: Ancestor, Environmental Stresses, and Antiseptics

**DOI:** 10.1128/mBio.02102-20

**Published:** 2020-12-15

**Authors:** Megha Sharma, Arunaloke Chakrabarti

**Affiliations:** a Department of Medical Microbiology, Postgraduate Institute of Medical Education and Research, Chandigarh, India; Westerdijk Fungal Biodiversity Institute; Leibniz Institute for Natural Product Research and Infection Biology - Hans Knoell Institute Jena (HKI)

**Keywords:** *Candida auris*, emerging infection, epidemiology, evolution

## Abstract

Candida auris has emerged as a serious threat to the health care settings. Advancements in molecular biology have provided several insights into the evolution of C. auris since it was first described in 2009. However, the simultaneous emergence of four different clades of the fungus at distinct geographical locations remains a mystery. The hypotheses already proposed by researchers fall short of explaining how and why C. auris emerged. In this article, we theorize that C. auris emerged from a common ancestor, subsequently migrated to specific geographical locations, and diversified genetically. This hypothesis is supported by genomic insights, historical events, and indirect scientific facts. C. auris adapted to humans at locations and times coinciding with the divergence from the most recent common ancestor, emerging almost simultaneously as an opportunist pathogen due to antiseptic practices. Future research will support or refute this hypothesis.

## Abstract (precis)

Candida auris has emerged as a serious threat to the health care settings. Advancements in molecular biology have provided several insights into the evolution of C. auris since it was first described in 2009.

## OPINION/HYPOTHESIS

The scientific community was unaware of the existence of Candida auris until 2009 when it was first reported as an agent of ear infection (1). Within the next decade, C. auris emerged as a “serious threat” to the health care settings and rapidly spread to more than 40 countries, resulting in multiple outbreaks with high mortality and multiple-drug resistance. Identification of the pathogen is difficult with routine diagnostic mycology, which adds to the problem (2). While we do not know the environmental niches of C. auris or how it has evolved as an opportunistic pathogen, we do know that this fungus commonly colonizes hospitalized patients (axilla, groin, and nares) (2). Nonetheless, definitive evidence of community transmission is lacking (3). To date, molecular analysis of global C. auris isolates have identified four distinct clades (clade I [South Asian], clade II [East Asian], clade III [South African], and clade IV [South American] [4, 5] with the possibility of a fifth clade from Iran [6]). The four distinct clades emerged almost simultaneously on different continents, but it is not known if all had a common ancestor (4).

Multiple hypotheses have been proposed to explain the emergence of C. auris. They include improved diagnostics (7), antifungal selection pressure (8), global warming (3), and human migration (9). Though each hypothesis addresses some aspects of the evolution of C. auris, none of them fully explain how and why C. auris emerged as a pathogen. The availability of molecular techniques has improved C. auris detection by overcoming the shortcomings of phenotypic identification. This alone cannot justify its emergence, as only six misidentified C. auris isolates were detected among 20,788 global *Candida* species isolates collected from 1997 to 2016 by the SENTRY Antifungal Surveillance Program (7). The theory that C. auris has been selected for by antifungal agents akin to azole-resistant Aspergillus fumigatus is also an insufficient explanation (8). The hot spots of C. auris do not coincide with areas of azole overuse or agriculture use. It also fails to account for the fitness costs associated with multidrug resistance (5). The third theory suggests that an overall increase in earth’s surface temperature allowed thermotolerant C. auris to breach the environment-to-human interface (3, 10). This first occurred in the rural areas possibly via an intermediate avian host, and subsequently to health care settings via human migration to urban cities. Climatic change has been linked to the emergence of new infectious diseases, including mycoses in general and C. auris infection in particular (11). However, unlike Candida glabrata with a reservoir in seabirds (12), C. auris has neither been isolated from any bird (13) nor do the routes of bird migration support the reported pattern of its clade distribution (14). Rural amplification also demands a proven community transmission (3). The human migration theory can justify coexistence of multiple clades at certain places (9), but it fails to clarify predominance of one clade at other places. Therefore, the origin of C. auris remains a mystery.

All theories proposed so far cannot explain the simultaneous emergence of different clades of C. auris around the world and its evolution as an opportunistic pathogen. Here we propose a hypothesis, connecting successive facts, which may explain the emergence of C. auris. We theorize the following: (i) There was a common ancestor of C. auris that was seeded into the areas where it has been reported (common ancestor). (ii) Significant environmental stresses coincided with divergence from the most recent common ancestor (TMRCA) and facilitated selection of each clade by breaching the environment-to-human interface (environmental stress). (iii) C. auris gained virulence and resistance traits while surviving silently on skin for years (stealth existence) and was selected for through the biased use of hand sanitizers in health care settings (antiseptic bias). We further propose that (iv) tsunamis played a role in the truncated evolution of clade II (tsunami-water cycling), thus contributing to the interclade differences.

### Common ancestor.

**FIG 1:**
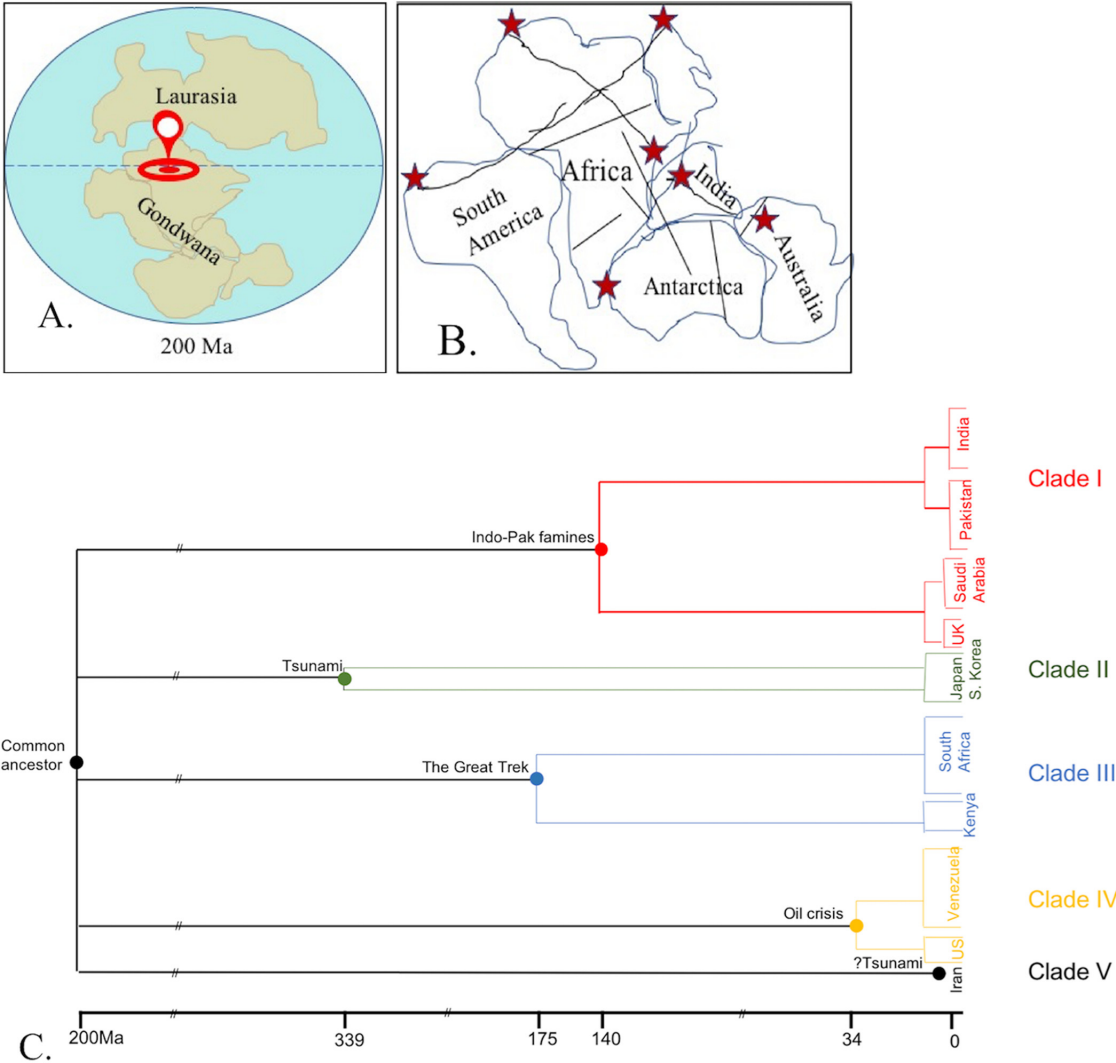
Diagrammatic representation of the possible location of the common ancestor of C. auris and the mechanism of its spread to specific geographical areas it emerged from. (a) The possible site of the common ancestor (as indicated by the red location pin) could be somewhere between the northern and central Sahara some 200 million years ago when the landmass of Earth consisted of Laurasia (Europe and North America) in the Northern Hemisphere and Gondwana (Africa, South America, India, Australia, and Antarctica) in the Southern Hemisphere. (b) The direction and extent of the major fault lineaments (indicated as interrupted lines), occurring secondary to tectonic activities of the Earth's crust, that could have served as possible routes for seeding of C. auris into the very regions it has been reported from (indicated by red stars). (c) Schematic timeline depicting the specific environmental disturbances coinciding with the most recent common ancestor of each clade of C. auris that could have precipitated the environment-to-human cross over of that clade at that particular place and time.

Several striking features, distinct from other *Candida* spp., raise the possibility of a common ancestor for the four clades of C. auris. However, the vast geographical distances between these clades questions the possible location of the common ancestor and its spread to distinct locations. Delving into the history of Earth, several missing links to the proposed common ancestor could be gathered. The Earth witnessed four major mass extinctions between 300 million years ago (Ma) and 170 Ma. The changing surface temperatures caused nearly all land species to go extinct, leaving behind few aquatic ecological niches where temperature changes were less steep (15). Molecular evolutionary studies of C. albicans isolates suggest that the *Candida* ancestor (possibly the ancestor of the CTG group containing C. albicans, C. tropicalis, C. auris) inhabited the Earth more than 800 Ma (16). It is possible that C. auris, forming part of an aquatic niche, survived through increasing temperatures (thermotolerance) via UV radiation-induced mutagenic changes (17) or overexpression of *HSP90* (5). So where could the reservoir of C. auris be? Around 250 Ma, the landmass of Earth consisted of a large continent named Pangea (meaning “all the landmass”) that later (∼200 Ma) separated into two halves due to tectonic activities: Gondwana (present-day Africa, South America, India-Pakistan, Australia, and Antarctica) in the south and Laurasia (present-day North America and Europe) in the north (Fig. 1a) (18). The emergence of C. auris at three specific sites (South Africa, India-Pakistan, and Venezuela) suggests that their common ancestor was located in Gondwana, possibly somewhere in northern Gondwana corresponding to modern-day Saharan Africa (Fig. 1a). Sahara became a desert some seven million years ago before which it had widespread wetlands and lakes, a possible ideal niche for C. auris. The mass extinctions in Gondwana may have given C. auris the opportunity to flourish (15), forming a part of one of the aquatic ecosystems that survived. The spread of C. auris may be explained by how Gondwana broke into different continents owing to tectonic activities some 150 million years ago. These tectonic activities led to major fault lineaments (linear zones of geological fractures or bends), especially between northern and central Africa (Fig. 1b), leading to an easy seepage of water as rivers, streams, and lakes (19). We believe these geological events may have transported water ecosystems containing C. auris to distant parts of Gondwana. Not only do these fault lines correspond to present-day rivers in India, Pakistan, and South Africa, but also the strategic locations of these faults correspond to the present-day sites of clade-specific C. auris distribution (i.e., Tibesti lineament extending from northern Africa touching Spain to Kenya for clade III, Guinean-Nubian and Levant lineaments extending from Venezuela to Israel for clade IV) (19). C. auris might have also lodged “along”’ the way, as is speculated for the region that escaped desertification (present-day Brazil), which harbors C. auris (20). Nevertheless, as the continents drifted apart from Gondwana, India-Pakistan attained its present-day location by merging into Asia, South America joined with North America, while Australia and Antarctica separated out to form independent continents (18). Japan could have also received a C. auris-containing aquatic niche from Gondwana, which is believed to have formed a small part of the island country (21). Hence, C. auris was “seeded” from the heart of Gondwana onto the very lands it has been documented from, i.e., India-Pakistan area (clade I), Japan (clade II), South Africa (clade III), and Venezuela (clade IV). Each clade was subsequently exposed to diverse environmental stresses over thousands of years leading to the differences seen today among the clades (5). The amplified fragment length polymorphism (AFLP)-derived minimum spanning tree placed African isolates of C. auris as a connecting link between closely related C. haemulonii and other clades (22). *C. haemulonii* was also isolated from a fish found in the Atlantic Ocean and seawater off the Portugal coast (5). These observations support the possible location of C. auris’ ancestor and its spread to the specific locations via these aquatic niches. We acknowledge that the great explorations of the Portuguese (23) during the 15th-17th centuries could have contributed to seeding of C. auris, as their travel itinerary coincides with the hot spots of South Africa, India, Australia, and Japan. However, this possibility does not support seeding of C. auris in Venezuela. Thus, our theory of the Gondwana common ancestor fits perfectly with how C. auris was seeded into specific geographical areas. However, its sudden emergence as a human pathogen in recent times is yet another mystery. To explain the sudden emergence, we propose that subsequent environmental stress has played an important role.

### Environmental stress.

Once seeded into the wetlands of these respective geographical areas, C. auris adapted to new ecological niches through various genetic and epigenetic changes. C. auris may have gained virulence and resistance traits from closely related species. Genomic studies revealed several orthologs of known virulence factors of C. albicans and *C. haemulonii* in C. auris (5, 24) and close relation to C. lusitaniae on functional annotation (25). The subsequent environmental stress explains how and when each C. auris clade breached the environment-to-human interface. The “molecular clock,” the measure of evolutionary change in nuclear material and diversification from related species over time, suggests that the time to the most recent common ancestor (TMRCA) was 140, 339, 175, and 34 years for clades I, II, III, and IV, respectively (9). Though the clade-specific ancestors simultaneously reached their particular geographic locations many years ago, certain environmental disbalances at particular times caused clonal expansion of each clade in their respective region (Fig. 1c). Exactly 140 years ago, between 1880 and 1890, India and Pakistan (one country at the time) faced three major famines (26), which led to gradual drying of bodies of water over large areas and subsequent intensive cultivation on the semidry river banks. This incident would have exposed some humans to wetlands harboring C. auris, thus introducing it onto human skin, maybe as a mere colonizer. Further, relief measures sought from mainland Britain and the nearest colonial port in Saudi Arabia (27) explains the common clade I in India, Pakistan, Britain, and Saudi Arabia. Regarding the emergence of clade II, major earthquakes in Japan (1677) and South Korea (1681) (28) and the subsequent tsunamis could have introduced C. auris from the Sea of Japan to mainland humans, as they coincide with the TMRCA of clade II. The possible transition of clade III from the environment to humans in South Africa occurred some 175 years ago. Surprisingly, it coincides with the Great Trek of Africa (29) involving massive human migration during the 1835s to the 1845s. Clade IV, having a TMRCA of just 34 years breached the environment-human barrier most recently. The emergence of C. auris in Venezuela is concomitant with desperate attempts to save its crippling economy by extracting oil from offshore fields around 1985 to 1989 (30). Such activities could have disrupted the aquatic ecological niches (possibly harboring C. auris). Similar environmental stress has been proposed for the emergence of Cryptococcus gattii in the Pacific Northwest. The passage of contaminated ballast water owing to opening of the Panama Canal coincides with the TMRCA of C. gattii (31). Though our hypothesis of environmental stress causing an environment-to-human transmission has no strong evidence to support it, it does have strong support of historical events and may fill the missing links of how each clade of C. auris gained entry into the human reservoir at their respective time and place.

### Stealth existence.

The question remains, if C. auris has been part of the human mycobiome all these years (34 to 339 years), why was it never reported earlier? It possibly had a stealth existence because of inadequacies of diagnostic techniques, lack of knowledge of the skin mycobiome, and absence of colonizing isolates in the collection of the SENTRY study (7). C. auris probably existed as a minute fraction of the skin mycobiome and even dispersed to involve nares and external ear. The question remains that if C. auris, fully equipped with resistance and virulence genes, was already surviving on human skin for many years in extremely small quantities, why did it suddenly create such a health care havoc? For this, we propose a possible explanation of antiseptic bias.

### Antiseptic bias.

Alcohol-based antiseptics perform best in the eradication of C. auris from skin (32, 33); however, their use is largely limited to the hands of health care workers as alcohol-based hand sanitizers and rubs. The body sites of patients are usually cleaned and decolonized using chlorhexidine, hydrogen peroxide, and non-alcohol-based antiseptics, which are not as effective against C. auris (32, 33), thus providing a growth opportunity for C. auris at these sites. One might argue that some resilience to these compounds is also exhibited by other species like C. albicans (32), so why is only C. auris biased by this? It is noteworthy that while planktonic stages of C. auris and C. albicans exhibit similar resilience to antiseptics, the biofilms of C. auris are much more resilient than those of C. albicans or C. glabrata to 0.5% chlorhexidine and 3% hydrogen peroxide (34). Surviving exposure to non-alcohol-based antiseptics and continuously being supplied from commensal sites (which were not exposed to hand sanitizers), C. auris might form biofilms and further replenish other colonizing sites. We believe that this antiseptic bias in the selection of antiseptic and choice of body site in the past decade has caused C. auris to emerge in health care settings and not as a community pathogen. Exposure to several broad-spectrum antimicrobials in critically ill patients may select for C. auris biofilms on skin, which can then gain access into the bloodstream via multiple invasive devices used in such patients.

The fact remains that unlike other clades, which cause invasive health care-associated infections and outbreaks, clade II is limited to infections of the ear. Clade II is also the least pathogenic and the least resistant clade despite being the oldest (35). We propose that the periodic tsunamis (>20 tsunamis in Japan; 3 in South Korea) since the TMRCA (28) might have brought the original seeded strains of C. auris to the mainland. Tsunamis have been implicated in increased fungal infections, be it invasive “tsunami lung” in a near-drowning Japanese patient (36) or emergence of Cryptococcus gattii in the Pacific Northwest (31). The ballast tank-tsunami hypothesis of C. gattii (31) gained popularity as the fungus was proven to be sexual where recombination was possible (37). Perhaps our theory of tsunami-water cycling will gain more impetus once a sexual phase is proven in C. auris. The observations that clade II has genomic relatedness to ancestral strains and lost subtelomeric sections responsible for adhesins (5) support the possibility of blunted evolution owing to local water cycling along the coasts. It is likely that a relatively low density of cells arrived on the mainland and they too were washed back into the sea via natural rain runoff or snow melt, thus hampering their coevolution with humans. Its access was limited to the external ear of divers and swimmers who entered such aquatic ecosystems and caused ear infection in the opportune host, like those on long-term antibiotics or with ear trauma (35). This theory of tsunami-coastal water cycling may be extrapolated to a C. auris-associated ear infection from Iran that has been proposed as the fifth clade (6). The initial primary seeding of C. auris in Iran remains enigmatic, and it may have been introduced into the Persian Gulf through trade and travel. The possibility exists that all four clades may have been introduced over time, allowing intra- and interclade transfer of genetic material (5). The increased number of tsunamis in Iran, occurring yearly since 2002 (38), may have caused rapid mixing of the old and new strains, thus giving rise to a strain similar to clade II in pathogenicity yet distinct enough to qualify as a novel clade. The fact that the 14-year-old Iranian patient (39) had no history of travel outside Iran but did have a history of frequent swimming suggests that she did not acquire C. auris from a distant land but that it was brought to her via water. The exact evolution of this fifth clade will become clearer as more isolates are subjected to molecular clock analysis.

C. auris, like a patient predator, had been lurking around from times immemorial. It kept adapting to the varied ecosystems that were presented to it, finally gaining access to the human host and causing the much-feared pathogenic switch.

### Hypothesis testing.

Future studies should aim to find evidence to prove or disprove our hypothesis. An attempt should be made to find the ancestral strains (from Sahara Desert lakes, lakes of Kenya, or wetlands of Brazil). A thorough whole-genome sequence-based comparison with contemporary strains would decipher the evolutionary attributes of different clades of C. auris and orthologs and paralogs within other *Candida* spp. Aggressive sampling of the wetlands and the skin mycobiome (especially targeting under nails, ear canal, nares) coupled with advanced deep sequencing techniques should be undertaken. Another study could compare the efficacy of alcohol and chlorhexidine in decolonizing patient bodies.

## References

[1] Satoh K, Makimura K, Hasumi Y, Nishiyama Y, Uchida K, Yamaguchi H. 2009. *Candida auris* sp. nov., a novel ascomycetous yeast isolated from the external ear canal of an inpatient in a Japanese hospital. Microbiol Immunol 53:41–44. doi:10.1111/j.1348-0421.2008.00083.x. 19161556

[2] Sabino R, Veríssimo C, Pereira ÁA, Antunes F. 2020. *Candida auris*, an agent of hospital-associated outbreaks: which challenging issues do we need to have in mind? Microorganisms 8:181. doi:10.3390/microorganisms8020181. PMC7074697 32012865

[3] Jackson BR, Chow N, Forsberg K, Litvintseva AP, Lockhart SR, Welsh R, Vallabhaneni S, Chiller T. 2019. On the origins of a species: what might explain the rise of *Candida auris*? J Fungi 5:58. doi:10.3390/jof5030058. PMC6787658 31284576

[4] Lockhart SR, Etienne KA, Vallabhaneni S, Farooqi J, Chowdhary A, Govender NP, Colombo AL, Calvo B, Cuomo CA, Desjardins CA, Berkow EL, Castanheira M, Magobo RE, Jabeen K, Asghar RJ, Meis JF, Jackson B, Chiller T, Litvintseva AP. 2017. Simultaneous emergence of multidrug resistant *Candida auris* on three continents confirmed by whole genome sequencing and epidemiological analyses. Clin Infect Dis 64:134–140. doi:10.1093/cid/ciw691. 27988485 PMC5215215

[5] Chybowska AD, Childers DS, Farrer RA, Barker BM. 2020. Nine things genomics can tell us about *Candida auris*. Front Genet 11:351. doi:10.3389/fgene.2020.00351. 32351544 PMC7174702

[6] Chow NA, De Groot T, Badali H, Abastabar M, Chiller TM, Meis JF. 2019. Potential fifth clade of *Candida auris*, Iran, 2018. Emerg Infect Dis 25:1780–1781. doi:10.3201/eid2509.190686. 31310230 PMC6711235

[7] Pfaller MA, Diekema DJ, Turnidge JD, Castanheira M, Jones RN. 2019. Twenty years of the SENTRY Antifungal Surveillance Program: results for *Candida* species from 1997–2016. Open Forum Infect Dis 6:S79–S94. doi:10.1093/ofid/ofy358. 30895218 PMC6419901

[8] Abdolrasouli A, Rhodes J, Beale MA, Hagen F, Rogers TR, Chowdhary A, Meis JF, Armstrong-James D, Fisher MC. 2015. Genomic context of azole resistance mutations in *Aspergillus fumigatus* determined using whole-genome sequencing. mBio 6:e00536-15. doi:10.1128/mBio.00536-15. 26037120 PMC4453006

[9] Chow NA, Munoz J, Gade L, Berkow EL, Li X, Welsh RM, Forsberg K, Lockhart SR, Adam R, Alanio A, Alastruey-Izquierdo A, Althawadi S, Arauz A, Ben-Ami R, Bharat A, Calvo B, Desnos-Ollivier M, Escandon P, Gardam D, Gunturu R, Heath CH, Kurzai O, Martin R, Litvintseva AP, Cuomo CA. 2020. Tracing the evolutionary history and global expansion of *Candida auris* using population genomic analyses. bioRxiv doi:10.1101/2020.01.06.896548. PMC7188998 32345637

[10] Casadevall A, Kontoyiannis DP, Robert V. 2019. On the emergence of *Candida auris*: climate change, azoles, swamps, and birds. mBio 10:e01397-19. doi:10.1128/mBio.01397-19. 31337723 PMC6650554

[11] Casadevall A. 2020. Climate change brings the specter of new infectious diseases. J Clin Invest 130:553–555. doi:10.1172/JCI135003. 31904588 PMC6994111

[12] Al-Yasiri MH, Normand A-C, L’Ollivier C, Lachaud L, Bourgeois N, Rebaudet S, Piarroux R, Mauffrey J-F, Ranque S. 2016. Opportunistic fungal pathogen *Candida glabrata* circulates between humans and yellow-legged gulls. Sci Rep 6:36157–36158. doi:10.1038/srep36157. 27782182 PMC5080578

[13] Cafarchia C, Iatta R, Danesi P, Camarda A, Capelli G, Otranto D. 2019. Yeasts isolated from cloacal swabs, feces, and eggs of laying hens. Med Mycol 57:340–345. doi:10.1093/mmy/myy026. 29762763

[14] BirdLife International. 2010. The flyways concept can help coordinate global efforts to conserve migratory birds. BirdLife International, Cambridge, United Kingdom.

[15] Button DJ, Lloyd GT, Ezcurra MD, Butler RJ. 2017. Mass extinctions drove increased global faunal cosmopolitanism on the supercontinent Pangaea. Nat Commun 8:733. doi:10.1038/s41467-017-00827-7. 29018290 PMC5635108

[16] Heckman DS, Geiser DM, Eidell BR, Stauffer RL, Kardos NL, Hedges SB. 2001. Molecular evidence for the early colonization of land by fungi and plants. Science 293:1129–1133. doi:10.1126/science.1061457. 11498589

[17] Bornman JF, Barnes PW, Robson TM, Robinson SA, Jansen MAK, Ballare CL, Flint SD. 2019. Linkages between stratospheric ozone, UV radiation and climate change and their implications for terrestrial ecosystems. Photochem Photobiol Sci 18:681–716. doi:10.1039/c8pp90061b. 30810560

[18] Torsvik TH, Cocks LRM. 2013. Gondwana from top to base in space and time. Gondwana Res 24:999–1030. doi:10.1016/j.gr.2013.06.012.

[19] Guiraud R, Bosworth W, Thierry J, Delplanque A. 2005. Phanerozoic geological evolution of Northern and Central Africa: an overview. J Afr Earth Sci 43:83–143. doi:10.1016/j.jafrearsci.2005.07.017.

[20] Pasqualotto AC, Sukiennik TCT, Meis JF. 2019. Brazil is so far free from *Candida auris*. Are we missing something? Braz J Infect Dis 23:149–150. doi:10.1016/j.bjid.2019.05.004. 31152687 PMC9428194

[21] Hada S, Ishii KI, Landis CA, Aitchison J, Yoshikura S. 2001. Kurosegawa terrane in southwest Japan: disrupted remnants of a Gondwana-derived terrane. Gondwana Res 4:27–38. doi:10.1016/S1342-937X(05)70652-3.

[22] Sarma S, Upadhyay S. 2017. Current perspective on emergence, diagnosis and drug resistance in *Candida auris*. Infect Drug Resist 10:155–165. doi:10.2147/IDR.S116229. 28652784 PMC5476417

[23] Barreto LF. 1989. Camöes and the Portuguese voyages of discovery. UNESCO Courier XLII 4:1–38. https://unesdoc.unesco.org/ark:/48223/pf0000083185. Last accessed on 15 October 2020.

[24] Muñoz JF, Gade L, Chow NA, Loparev VN, Juieng P, Berkow EL, Farrer RA, Litvintseva AP, Cuomo CA. 2018. Genomic insights into multidrug-resistance, mating and virulence in *Candida auris* and related emerging species. Nat Commun 9:5346. doi:10.1038/s41467-018-07779-6. 30559369 PMC6297351

[25] Chatterjee S, Alampalli SV, Nageshan RK, Chettiar ST, Joshi S, Tatu US. 2015. Draft genome of a commonly misdiagnosed multidrug resistant pathogen *Candida auris*. BMC Genomics 16:686. doi:10.1186/s12864-015-1863-z. 26346253 PMC4562351

[26] Purkait P, Kumar N, Sahani R, Mukherjee S. 2020. Major famines in India during British rule: a referral map. Available at https://www.researchgate.net/publication/340224385_Major_Famines_in_India_during_British_Rule_A_Referral_Map. Last accessed on 15 October 2020.

[27] Ochsenwald WL, Philby HSJB, Teitelbaum J. 2020. Saudi Arabia. Encyclopedia Britannica Inc, Chicago, IL.

[28] Hatori T. 1975. Tsunami source off Boso peninsula − estimation of tsunami source area and magnitude of Enpo (1677), Genroku (1703) and 1953 Boso-oki tsunami. Bull Earthq Res Inst 50:83–91.

[29] The Editors of Encyclopaedia Britannica. 2020. Great Trek − South African history. Encyclopedia Britannica Inc, Chicago, IL.

[30] Aljazeera News. 3 May 2017. Venezuela’s worst economic crisis: what went wrong? Al Jazeera. https://www.aljazeera.com/features/2017/5/3/venezuelas-worst-economic-crisis-what-went-wrong.

[31] Engelthaler DM, Casadevall A. 2019. On the emergence of *Cryptococcus gattii* in the Pacific Northwest: ballast tanks, tsunamis, and black swans. mBio 10:e02193-19. doi:10.1128/mBio.02193-19. 31575770 PMC6775458

[32] Fu L, Le T, Liu Z, Wang L, Guo H, Yang J, Chen Q, Hu J. 2020. Different efficacies of common disinfection methods against *Candida auris* and other *Candida* species. J Infect Public Health 13:730–736. doi:10.1016/j.jiph.2020.01.008. 32005617

[33] Rutala WA, Kanamori H, Gergen MF, Sickbert-Bennett EE, Weber DJ. 2019. Susceptibility of *Candida auris* and *Candida albicans* to 21 germicides used in healthcare facilities. Infect Control Hosp Epidemiol 40:380–382. doi:10.1017/ice.2019.1. 30767810

[34] Kean R, McKloud E, Townsend EM, Sherry L, Delaney C, Jones BL, Williams C, Ramage G. 2018. The comparative efficacy of antiseptics against *Candida auris* biofilms. Int J Antimicrob Agents 52:673–677. doi:10.1016/j.ijantimicag.2018.05.007. 29775686

[35] Kim M, Shin JH, Sung H, Lee K, Kim E, Ryoo N, Lee J, Jung S, Park KH, Kee SJ, Kim SH, Shin MG, Suh SP, Ryang DW. 2009. *Candida haemulonii* and closely related species at 5 university hospitals in Korea: identification, antifungal susceptibility, and clinical features. Clin Infect Dis 48:e57–e61. doi:10.1086/597108. 19193113

[36] Nakamura Y, Suzuki N, Nakajima Y, Utsumi Y, Murata O, Nagashima H, Saito H, Sasaki N, Fujimura I, Ogino Y, Kato K, Terayama Y, Miyamoto S, Yarita K, Kamei K, Nakadate T, Endo S, Shibuya K, Yamauchi K. 2013. *Scedosporium aurantiacum* brain abscess after near-drowning in a survivor of a tsunami in Japan. Respir Invest 51:207–211. doi:10.1016/j.resinv.2013.07.001. 24238227

[37] Phadke SS, Feretzaki M, Clancey SA, Mueller O, Heitman J. 2014. Unisexual reproduction of *Cryptococcus gattii*. PLoS One 9:e111089. doi:10.1371/journal.pone.0111089. 25337713 PMC4206507

[38] Heidarzadeh M, Pirooz MD, Zaker NH, Mokhtari M. 2007. Evaluating the tsunami hazard in the Persian Gulf and its possible effects on coastal regions. Available at https://www.academia.edu/15023424/Evaluating_the_Tsunami_Hazard_in_the_Persian_Gulf_and_its_Possible_Effects_on_Coastal_Regions?ssrv=c. Last accessed on 15 October 2020.

[39] Abastabar M, Haghani I, Ahangarkani F, Rezai MS, Taghizadeh Armaki M, Roodgari S, Kiakojuri K, Al-Hatmi AMS, Meis JF, Badali H. 2019. *Candida auris* otomycosis in Iran and review of recent literature. Mycoses 62:101–105. doi:10.1111/myc.12886. 30585653

